# Pelvic floor complaint profiles in young adult women; a file review study in pelvic physical therapy practices in the Netherlands

**DOI:** 10.12688/openreseurope.19785.1

**Published:** 2025-03-24

**Authors:** Alma Brand, Wim Waterink, Xynthia Kavelaars, Jacques van Lankveld

**Affiliations:** 1 Faculty of Psychology, Open University of The Netherlands, Heerlen, The Netherlands

**Keywords:** pelvic floor complaints, complaint profiles, latent class analysis

## Abstract

**Background:**

Many patients with pelvic floor complaints, including urinary and fecal incontinence, micturition and defecation problems, pelvic organ prolapses, pelvic pain, and painful intercourse, receive pelvic physical therapy treatment. Based on available evidence, pelvic floor complaint profiles, including these complaints, have not been investigated but may help to enhance therapists’ clinical reasoning and patients’ understanding and self-disclosure.

**Aim:**

This retrospective file review study explores preliminary complaint profiles and associations between pelvic floor complaints.

**Method:**

Pelvic physical therapists entered recorded pelvic floor complaints from self-selected pregnant, parous, and nulliparous patients’ files in an online survey. Descriptive statistics and correlations were calculated, and latent class analysis was performed to gain insight into pelvic floor complaint profiles and their associations with pregnancy and parity.

**Results:**

A model with five profiles was selected based on statistical and theoretical selection criteria. One profile was characterized by pelvic pain, one by defecation and micturition problems, one by fecal incontinence and defecation problems, another by pelvic organ prolapses and urinary incontinence, and one by painful intercourse and micturition problems. The first and second profiles appeared most characteristic for pregnant, the third and fourth for parous, and the fifth for nulliparous patients.

**Conclusion:**

The identified profiles may facilitate the inclusion and understanding of etiological factors for combined pelvic floor complaints in clinical practice and scientific research. Addressing pelvic floor complaints in profiles may help pelvic healthcare providers during their history-taking, enhance multidisciplinary treatment approaches, and help patients understand experienced combinations of pelvic floor complaints. This may ultimately benefit women’s pelvic health.

## Plain Language Summary

Many patients with pelvic floor complaints, such as urinary and fecal incontinence, bladder and bowel issues, pelvic organ prolapses, pelvic pain, and painful intercourse, receive pelvic physical therapy treatment. To date, profiles of pelvic floor complaints, including these conditions, have not been investigated. In a retrospective file review study, pelvic floor complaint profiles have been examined. For this purpose, pelvic physical therapists entered documented pelvic floor complaints from self-selected files of pregnant patients and patients with or without children in an online survey. Various statistical analyses were conducted to gain insight into the pelvic floor complaint profiles and their associations with pregnancy and parity. Five clinically relevant complaint profiles emerged. Two profiles, one with a high likelihood of pelvic pain and the other of defecation and micturition problems, appeared typical for pregnant patients. Two profiles, one featuring a high likelihood of fecal incontinence and defecation issues and the other of pelvic organ prolapses and urinary incontinence, were characteristic of patients with children. The fifth profile, which included a high likelihood of painful intercourse and micturition problems, seemed to be typical for patients without children. The identified profiles may enhance understanding of the causes for combinations of pelvic floor complaints, which could be useful in clinical practice and scientific research. Addressing pelvic floor complaints through these profiles may aid pelvic healthcare providers during their history-taking, enhance multidisciplinary treatment approaches, and assist patients in understanding the combinations of pelvic floor complaints they experience. This may ultimately benefit women’s pelvic health.

## Introduction

Pelvic physical therapists (PTs) receive training in diagnosing, evaluating, and treating pelvic floor complaints (PFCs) such as urinary and fecal incontinence, micturition and defecation problems, pelvic organ prolapses, pelvic pain, and painful intercourse that patients experience in their daily, social, and sexual functioning and their intimate relationships ^1– 5^. Ideally, PTs address all common PFCs in their history-taking to obtain a comprehensive picture of the patient’s pelvic health status. This and other information collected helps PTs in their clinical reasoning to reach a working diagnosis. Based on the obtained diagnosis, necessary referrals can be made, psychoeducation can be provided, and treatment approaches can be selected ^6, 7^. The latter could be multidisciplinary, including medical specialists, psychologists, and sexologists.

However, a higher-than-desirable focus on isolated complaints exists in research and clinical pelvic physical therapy (PPT) practice ^8– 10^. The literature recommends rather than indicates the consideration of addressing multiple PFCs, such as asking about micturition and defecation problems in women with pelvic organ prolapses ^11^. Insurance companies may require a diagnosis to be coded based on the most prominent PFC, which may increase the risk of narrowing the diagnostic scope, excluding less prominent PFCs from the treatment approach. An undesirable focus on only the main PFC in, for example, a patient’s daily functioning may result in unsatisfactory treatment outcomes when the impact of this main complaint and potentially co-occurring PFCs on, for instance, the patient’s social or sexual functioning remains undertreated ^11^. A more concrete example would be ignoring or undertreating a defecation problem such as constipation in patients with pelvic organ prolapses, leaving them to push hard to evacuate stool, which could result in an unsatisfactory treatment outcome, excluding this risk- or maintaining factor to pelvic organ prolapse from the treatment approach ^12^.

Instead, thinking and reasoning regarding commonly prevalent PFC complaints in PFC profiles might help increase scientific and clinical insight into and attention to underlying etiological predisposing, precipitating, or maintaining factors of various (common) PFC combinations. A comprehensive reasoning strategy is theoretically relevant since it might facilitate research into understanding the causes, prognoses, and challenges of different presentations of PFCs. Moreover, comprehensive reasoning regarding prevalent combinations of PFCs in PFC profiles is clinically relevant as it may reduce the risk of overlooking, ignoring, or excluding additional PFCs crucial for improving patients’ pelvic health. In clinical practice, comprehensive reasoning has the potential to complement psychoeducation content and ultimately enhance treatment (multidisciplinary) approaches and results. In addition, patients may experience additional benefits from better insight into and understanding of causes, prognoses, and combinations of PFCs, which could facilitate their self-disclosure of PFCs that may have been appraised as irrelevant or unrelated to their problems.

Well-known etiological factors of PFCs are pregnancy, the number of births, delivery mode, and extent of PFM damage during childbirth ^4, 7, 13– 19^. Due to their different nature, distinguishing between pregnant, parous, and nulliparous patients is important. Identifying PFC profiles in these groups of patients might enhance understanding of risk factors for specific PFC combinations in these patient groups. In addition, the nature and severity of PFCs appear to co-occur with hormonal fluctuations during women’s menstrual cycle ^20^. More insight into the complex association between these hormone-related factors and PFCs might help comprehend their association and impact on women and enhance treatment options and symptom relief ^20^. More research cues may be generated, and additional research may be needed to improve understanding of the association between hormonal contributing or causal factors to PFCs. Therefore, this exploratory study aimed to identify clinically relevant PFC profiles that may contribute to including predisposing, precipitating, and maintaining factors in scientific research and enhance clinical reasoning in PPT practice. In addition, the associations between PFCs and menstrual complaints were explored to gain more insight into the contributing character of the menstrual cycle to specific PFCs.

## Methods

### Ethical considerations

The Ethical Review Board of the Open University of the Netherlands approved the study protocol (Date: May 29th, 2019/No. U2019/03973/HVM) in May 2019. Before participation, PTs and patients whose files were selected to be reviewed provided informed consent.

### Design and procedure

An exploratory retrospective file review study was conducted among Dutch PTs. The data were anonymously collected in an online survey using the online data acquisition portal O4U ( https://o4u.ou.nl/en/node/234). The principal investigator (AB) and a PT pilot-tested the survey’s functionality. Their test data indicated a need to adapt two minor interpretation issues, after which the survey was retested by two other PTs, who did not identify additional issues.

PTs received a link to access the information letter explaining the study’s background and objectives on the online study platform. Informed consent forms for patients could be downloaded or sent by regular mail on request. After signing the online informed consent form, PTs completed a brief questionnaire concerning their qualifications, registration, employment status, work experience as PT, and workplace settings, including private practices, hospitals, healthcare centers, nursing homes, and rehabilitation centers. PTs were then instructed to enter anonymous data from up to 30 patient files into identical online questionnaires. The signed informed consent forms from the selected patients were sent to the principal investigator and stored in compliance with applicable privacy laws and regulations. Data entry took a few minutes per patient. After conducting a sensitivity analysis, showing the absence of differences between preliminary test data and data collected in the operational phase, the test data were added to the final dataset to increase the number of included patient files and the ensuing robustness of the results.

### Participants

**PT inclusion and recruitment:** PTs interested in participating were included when they were registered, qualified to perform internal vaginal and anal examinations, and currently practicing in the Netherlands. The two Dutch PPT societies assisted with the recruitment of PTs through their newsletters. PTs were also recruited verbally and online via LinkedIn and Facebook. Society members were rewarded for participation with (compulsory) permanent education credits for continued registration in the Dutch PPT quality registers.

**Patient file selection:** PTs were instructed to include files from pregnant patients who were expecting their first child, parous patients who had given birth to their youngest child no more than two years ago and were not pregnant again, and nulliparous patients who did not have children and were not pregnant. All patients were aged between 18 and 45. The criteria were chosen to obtain the purest profiles concerning pregnancy and parity. The selected patients were preferably treated at the time of participation or within the past year.

Each PT was initially asked to document data from ten pregnant, ten parous, and ten nulliparous patients to obtain equally sized subgroups, assuming every PT would easily find ten women from each group in their caseload. When participating PTs immediately labeled this request challenging due to their varying caseloads and PT specializations, registering ten patients per group was changed to preferable but not compulsory. Due to the stratified sampling procedure and the specializations of PTs, the distribution of patients and their characteristics in the sample may differ from the distribution in the target population.

### Measurement instrument

The requested data included age, pregnancy- and parity status, types of PFCs, and menstrual complaints as recorded in the patient file. For pregnant patients, gestation age in months and the number of miscarriages were recorded. The number of children, the age of the youngest child, and the number of vaginal deliveries and cesarian sections were inventoried for parous patients.

Based on the PT Society coding list, a reference catalog was compiled to clarify and define symptoms and guide PTs in labeling and documenting PFCs online using the same definitions and categories. The selection of PFCs included in the catalog was based on the expert opinions and consensus of five randomly chosen PTs. These PTs strongly advised including menstrual complaints in the coding list because of their prevalence among patients (see Table 1). Two independent and randomly chosen PTs pilot-tested the catalog and approved the content and logic.

**Table 1.** Reference catalog of the pelvic floor and menstrual complaint symptoms.

Catalog items		Symptoms and Definitions	NVFB Diagnoses	Categorized PFC
Pelvic Floor Complaints (PFC)			
**1**	**Urinary** **Incontinence**	Involuntary loss of urine when sneezing, coughing, laughing, jumping etc., Urge incontinence, Loss of urine during sex, Unnoticed loss of urine, or Leakage after micturition	Stress Urinary Incontinence (SUI) Urge Urinary Incontinence (UUI) Mixed Urinary Incontinence (MUI)	**Urinary** **Incontinence**
**2**	**Fecal Incontinence**	Involuntary loss of feces, Unnoticed loss of feces, Involuntary loss of feces when sneezing, coughing, laughing, jumping etc.	Fecal Incontinence Soiling Stamping	**Fecal Incontinence**
**3**	**Flatus**	Inability to hold wind, Excessive intestinal gas formation and bother of flatus, Flatus related to specific types of food	Anal Flatus	
**4**	**Dysfunctional Voiding**	Hesitation, Incomplete voiding, Pushing to urinate, Changed flow, Postponing micturition, Not taking time for micturition	Dysfunctional Voiding Urinary Retention	**Micturition Problems**
**5**	**Urge / Frequency (micturition)**	Frequent micturition, Frequent urge, Imperative urge, Painful urge, Interstitial Cystitis	Frequency Interstitial Cystitis Overactive Bladder	
**6**	**Urinary Tract Infections**	Burning pain during micturition, Loss of control over urge, Sense of pressure in/on bladder, Frequent urge	Urinary Tract Infections	
**7**	**Urge / Frequency** **(defecation)**	Empty insistence, Incomplete emptying, Frequent urge, Difficulty postponing urge	Fecal Urge Frequency	**Defecation Problems**
**8**	**Constipation**	Having to push (hard) to empty bowel, Feeling bloated or full, Abdominal pain because of full bowel, No appetite, Postponing defecation, Overflow	Constipation Slow Transit Spastic Colon	
**9**	**Anal Complaints**	Hemorrhoids, Fissures, Pain during defecation, Anal cramp	Anal Cramp Anal Fissures	
**10**	**Vaginal Pelvic Organ Prolapse**	Sense of vaginal pressure, Heaviness, Sense of ball protruding, Visible ball protruding, Tampons do not stay put, Cystocele, Rectocele, Uterus prolapse	Cystocele Descensus Uteri Prolapse Rectocele	**Pelvic Organ** ** Prolapse**
**11**	**Rectal Pelvic Organ Prolapse**	Sense of anal pressure, Protrusion of rectum through anus	Enterocele Prolapse	
**12**	**Low Back / Pelvic Pain**	Low back pain, Hernia, Pelvic pain, SI-joint pain, Buttock pain, Groin pain, Hip problems, Pain that spreads into the legs, Starting stiffness	Low Back Pain Pelvic Pain Pregnancy-related Pelvic Pain (pre- and postnatal) (Sub)Luxation	**Pelvic Pain**
**13**	**Genital Pain**	Perineal pain, Vulvodynia, Painful scars	Pelvic Floor Pain Scar Tissue Lacerations	
**14**	**Coccyx Pain**	Coccyx pain during sitting, sitting down and standing up from sitting	Coccygodynia	
**15**	**Painful Intercourse**	Penetration pain, Inability to use tampons, Inability to insert a finger in vagina, Vaginismus, Deep vaginal pain during penetration sex, Orgasm problems resulting from pelvic floor muscle tension	Dyspareunia Vaginismus Vulvar Pain Syndrome Vulvar Vestibulitis Syndrome	**Painful Intercourse**
**16**	**Menstrual Complaints**	Increase of pelvic floor complaints prior to and/or during the first days of menstruation		**Menstrual** **Complaints**

PTs were instructed to document the recorded PFCs and menstrual complaints from the patient files included in the catalog and according to the catalog’s descriptions. First, PTs were instructed to document the complaint as stated during registration for treatment. Then, they were instructed to document the most bothersome complaint because the registration complaint may not necessarily be experienced as such. Finally, they documented all noted complaints from the patient files.

### Data-analysis

Data analysis was conducted using SPSS-28 ^21^, Excel, and R ^22^. To improve clarity, the 15 PFC symptoms from the catalog were merged into the seven common PFCs, as indicated in the right column of Table 1 before analysis ^16^. PFCs and menstrual complaints were labeled as present with a score of ‘1’ when one or more symptoms from the catalog were recorded and a score of ‘0’ when no symptoms were recorded. This scoring procedure produced a data set of binary outcome variables.

First, descriptive statistics were calculated in SPSS and Excel, encompassing the recorded frequencies and percentages of the types of pelvic floor and menstrual complaints. The statistics were calculated for the total sample and subgroups of pregnant, parous, and nulliparous patients. In addition, pairwise correlations between the proportions of all outcome variables were computed. To account for their dichotomous measurement level, pairwise correlations were computed using the following formula: r = (y(11) – y1*y2)/(y1*(1-y1)*y2*(1-y2))^(1/2) ^23^. In this formula, y(11) denotes the proportion of patients having both complaints recorded. The proportion of patients for whom complaint ‘1’ was recorded is indicated by y1, and y2 reflects the proportion of patients for whom complaint ‘2’ was recorded.

Latent class analysis (LCA) was used to identify prevalent combinations of three or more PFCs in PFC profiles. Data from all included patient files were used to cluster patients given any likelihood of typical pregnant, parous, and nulliparous patient profiles. A sample size of between 300 and 1000 respondents is needed to obtain reliable LCA outcomes ^24^. The LCA model selection procedure was based on statistical and theoretical considerations ^25^. Statistically, log-likelihood indices and the Bayesian Information Criterion (BIC) were used to select plausible numbers of patient classes (see extended data Appendix A). Three expert PTs were consulted to verify the principal researcher’s clinical interpretation and relevance of the emergent LCA models based on their experiences in PPT practice.

## Results

### Descriptive results

Twenty-two PTs who worked in private practices submitted data. Three also worked in a hospital or healthcare center. Data from 366 patients were analyzed. The final percentages in each patient group did not reflect the initially intended equally large groups of pregnant, parous, and nulliparous patients. A remarkable observation concerned recorded menstrual complaints for some pregnant patients. These menstrual complaints may have been recorded during the treatment of this patient before the pregnancy because menstruation is unlikely during pregnancy. The demographic information concerning the PTs and patients is provided in Table 2.

**Table 2.** Background information about the therapists and the selected patients.

Participants	Pelvic Physical therapists	Selected patients	Pregnant patients	Parous patients		Nulliparous patients
**N**	22	366	76 (20.8%)	205 (56.0%)		85 (23.2%)
**Average nr. of years** **of PPT experience**	12.59 (SD = 6.48)					
**Average nr. of patients included**	16.64 (SD = 11.57)					
**Mean age in years**		31.31 (SD = 5.54)	30.11 (SD = 3.68)	32.83 (SD = 4.11)		28.69 (SD = 8.17)
**Mean gestation age In weeks**			23.59 (SD = 7.64)			
				**Nr of children per patient**	**Patients with x nr. of children**	
				1 2 3 4 5	114 64 21 5 1	
**Average nr. of children (N = 194)**				1.61 (SD = 0.81)		
				**Age group**	**Nr. of children per age group**	
				0-6 months 6-12 months 12-18 months 18-24 months	111 36 24 23 **Total: 194**	
**Mean age of children in months**				7.74 (SD = 6.84)		

Table 3 presents the percentages of the recorded PFC at (self)referral in the left column, the most bothersome PFC during the intake in the middle column, and all recorded PFCs in the files in the right column. Pelvic pain was the most frequently recorded PFC and the PFC for which most patients registered for PPT. Pelvic pain and defecation problems were the most frequently recorded PFCs for pregnant patients. Urinary and fecal incontinence and pelvic organ prolapses were most frequently recorded for parous patients. Micturition problems, painful intercourse, and menstrual complaints were the most frequently recorded complaints for nulliparous patients.

**Table 3.** Distribution of pelvic floor and menstrual complaints in the patient files.

Recorded Complaints	Group	Percentage of the recorded complaint at referral	Percentage of the most bothersome complaint during intake	Percentage of all the recorded PFCs in the patient files
Pelvic Floor Complaint (PFC)		%	%	%
**Urinary Incontinence (UI)**	Total Sample	18.3	15.3	34.2
	Pregnant women	3.9	5.3	22.4
	**Parous women**	**27.3**	**22.4**	**44.9**
	Nulliparous women	9.4	7.1	18.8
**Fecal Incontinence (FI)**	Total Sample	2.2	2.5	5.5
	Pregnant women	0.0	0.0	1.3
	**Parous women**	**3.4**	**4.4**	**8.8**
	Nulliparous women	1.2	0.0	1.2
**Micturition Problems (MP)**	Total Sample	9.5	11.4	35.8
	Pregnant women	9.2	11.8	35.5
	Parous women	6.4	7.8	27.8
	**Nulliparous women**	**17.6**	**20.0**	**55.3**
**Defecation Problems (DP)**	Total Sample	4.1	7.1	30.9
	**Pregnant women**	1.3	6.6	**40.8**
	Parous women	2.5	5.4	25.9
	Nulliparous women	**10.6**	**11.8**	34.1
**Pelvic Organ Prolapse (POP)**	Total Sample	10.6	10.9	19.7
	Pregnant women	1.3	2.6	5.3
	**Parous women**	**18.1**	**17.6**	**31.7**
	Nulliparous women	1.2	2.4	3.5
**Pelvic Pain (PP)**	**Total Sample**	**58.4**	**57.9**	**73.0**
	**Pregnant women**	**85.5**	**84.2**	**89.5**
	Parous women	60.5	57.6	67.8
	Nulliparous women	29.4	33.0	70.6
**Painful Intercourse (PI)**	Total Sample	11.7	12.6	23.5
	Pregnant women	2.6	2.6	9.2
	Parous women	7.3	8.3	18.5
	**Nulliparous women**	**30.6**	**31.8**	**48.2**
**Menstrual Complaints (MC)**	Total Sample	0.5	1.4	7.4
	Pregnant women	0.0	0.0	1.3
	Parous women	0.5	0.5	4.9
	**Nulliparous women**	**1.2**	**4.7**	**18.8**

Note: N total sample = 366, N pregnant women = 76, N parous women= 205, N nulliparous women = 85. Per complaint, the subgroup with the highest percentages is highlighted in bold.

### Bivariate correlations

Table 4 shows the pairwise correlations between PFCs and menstrual complaints. Positive correlations indicate the probability of complaints (not) being recorded together, and negative correlations indicate the probability of one complaint being recorded but not the other. Fecal incontinence and defecation problems were the most frequently recorded pair of PFCs. Painful intercourse and menstrual complaints were most frequently recorded together. The negative correlations between fecal incontinence and pelvic pain implied that it is more likely to have either complaint recorded than to have both recorded.

**Table 4.** Pairwise correlations between pelvic floor and menstrual complaints.

	UI	FI	MP	DP	POP	PP	PI
Urinary Incontinence: **UI**	-						
Fecal Incontinence: **FI**	**0.08**						
Micturition Problems: **MP**	**0.05**	-0.03					
Defecation Problems: **DP**	-0.01	**0.15**	**0.06**				
Pelvic Organ Prolapse: **POP**	**0.01**	**0.00**	**0.00**	-0.03			
Pelvic Pain: **PP**	-0.08	**-0.14**	-0.01	-0.01	-0.07		
Painful Intercourse: **PI**	**0.01**	-0.05	**0.13**	**0.02**	-0.01	-0.08	
Menstrual Complaints: **MC**	**0.00**	-0.07	**0.05**	**0.01**	-0.06	**0.07**	**0.20**

Note: **N = 366** In Bold, positive correlations are highlighted. In Red, the positive correlations higher than 0.10 are indicated. In Blue, the negative correlations lower than 0.10 are indicated.

### Complaint profiles

**Figure 1.**
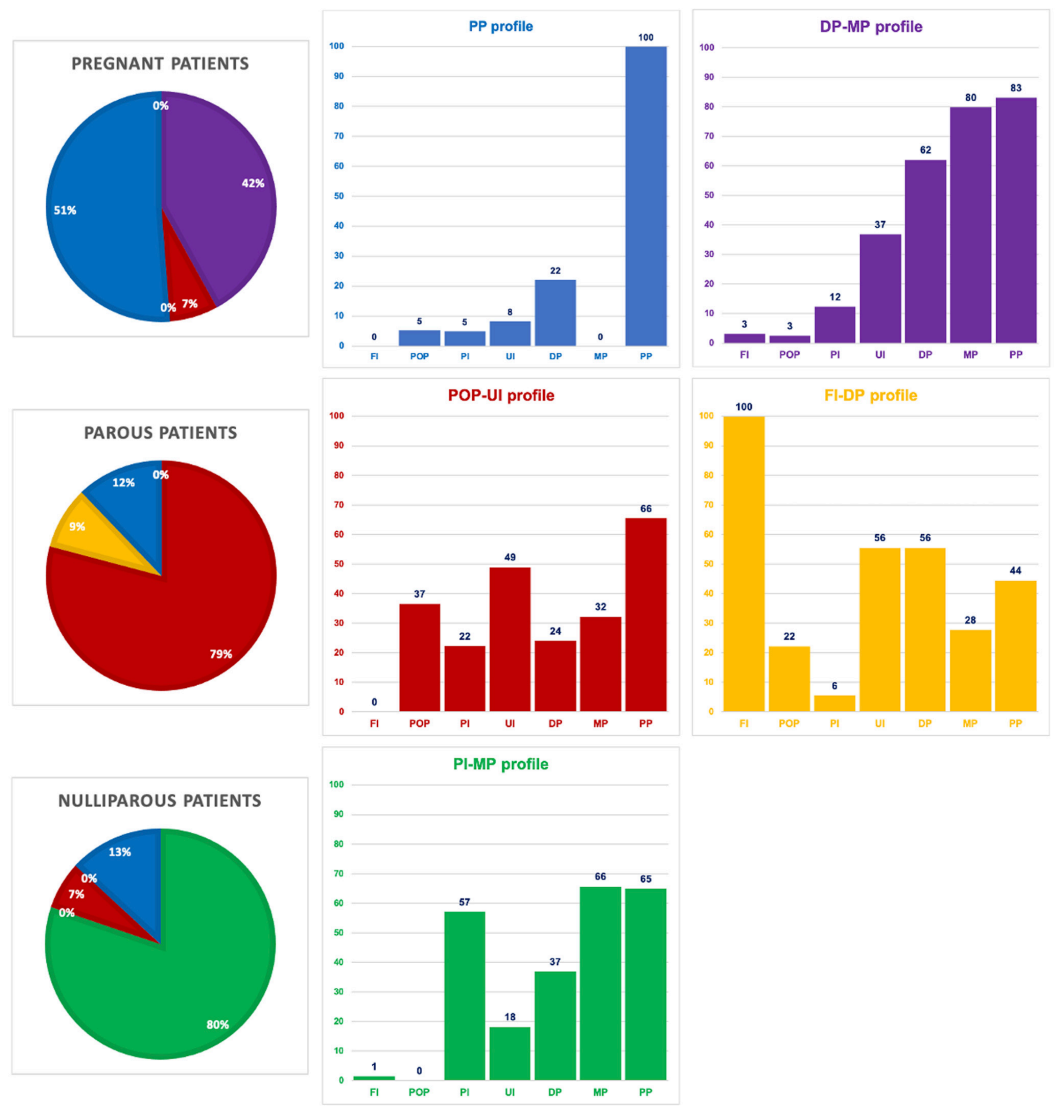
Five PFC profiles, including the likelihood of present PFCs within the profiles and the likelihood of each profile in each patient group. Note: FI = Fecal incontinence, POP = Pelvic organ prolapse, PI = Painful intercourse, UI = Urinary incontinence, DP = Defecation problems, MP = Micturition problems, PP = Pelvic pain. The bar charts reflect the likelihood in % of each complaint to be present in a profile. On the left, the probability of the presence of each profile in pregnant, parous, and nulliparous patient files is shown.

The stepwise LCA model selection process is explained in the extended data (see Appendix A). Based on the LCA selection criteria, the selected single-level model included five statistically plausible and clinically relevant PFC profiles. The probabilities of PFCs being present in the profiles are presented in Figure 1. Similarly, the accompanying pie charts indicate the probabilities for each PFC profile to be recorded in pregnant, parous, and nulliparous patients’ files. Although the five profiles were named for clarity after the most relevant PFCs in each profile, the PFCs with lower probabilities are not to be disqualified.

Two profiles, the blue profile with pelvic pain as the main complaint and the purple profile with defecation and micturition problems as the main complaints, appeared most characteristic for pregnant patients. Two profiles, the red profile with pelvic organ prolapses and urinary incontinence as the main complaints, and the yellow profile with fecal incontinence and defecation problems as the main complaints, appeared most characteristic for parous patients. The green profile, with painful intercourse and micturition problems as the main complaints, appeared most characteristic for nulliparous patients. The higher than 50% probability of recorded pelvic pain in four profiles classifies this complaint as a prominent feature treated in PPT practice. The five PFC profiles were found clinically relevant based on the experiences of the three consulted experts and the principal researcher. They recognized the combinations of main complaints in the profiles and frequently encountered them in each patient group.

## Discussion

### Complaint profiles

This study explored PFC profiles based on data from patient files in PPT practice, connecting these to pregnancy and parity status. Two profiles, the (blue) pelvic pain profile and the (purple) defecation and micturition problems profile, seem mostly related to pregnancy. The defecation and micturition problems profile also includes a high probability of pelvic pain. This is not surprising since pelvic pain is found to be a common complaint in pregnant women ^26, 27^. Although pelvic pain is the common denominator, it is not an isolated PFC. These two profiles show substantial differences in the likelihood of present urinary incontinence, defecation- and micturition problems, and, to a lesser extent, painful intercourse. These findings indicate the need for more research on potential risk factors, such as the number of miscarriages and connective tissue conditions, which may clarify the differences in probabilities of prevalent PFCs in both profiles ^28, 29^. Future research may help unravel etiological factors that help predict which pregnant patients may develop which combinations of PFCs based on the profiles and help choose adequate treatment approaches.

 Fecal incontinence and pelvic organ prolapses were less likely to be found among pregnant patients and seemed mostly related to childbirth. This is demonstrated by the (yellow) fecal incontinence and defecation problems and (red) pelvic organ prolapse and urinary incontinence profiles, which were more diffuse and characteristic of parous patients. Again, these findings were unsurprising, given that childbirth increases the risk of experiencing the main PFCs in the profiles postpartum ^15, 30^. Fecal incontinence and defecation problems are found to be common PFCs after (sub)total lacerations of the perineum and anal sphincter during vaginal deliveries ^31, 32^. Pelvic organ prolapses and urinary incontinence are primarily seen among parous patients after vaginal deliveries with prolonged pressure on and stretching of the vaginal connective tissue and bladder neck ^30, 33^. These heterogeneous profiles present a more diffuse range of PFCs, with lower probability peaks except for fecal incontinence, which predominantly distinguishes both profiles. Whether these profiles include sub-profiles related to previous deliveries and delivery circumstances is unclear. Future research could focus on identifying potential sub-profiles within a larger sample of parous patients, including potential etiological factors, such as the types and number of previous births, the extent of pelvic floor (muscle) damage, and the post-partum term of recovery after childbirth, which might help to explain the observed heterogeneity in the yellow and red profiles.

The fifth (green) painful intercourse and micturition problems profile appeared to be the most characteristic of nulliparous patients. This finding is also not surprising because painful intercourse and micturition problems are more often encountered together in nulliparous women ^34^. Although both main complaints are the common denominator in this profile, they are not exclusive of PFCs in nulliparous patients. The green profile shows that pelvic pain is also likely to be experienced by nulliparous women and, to a lesser extent, defecation problems and urinary incontinence. Excluding the latter PFCs from clinical reasoning and choosing treatment approaches may jeopardize treatment results and satisfaction in this group of patients.

The five profiles appear scientifically and clinically relevant and add to our understanding of PFC profiles concerning pregnancy and childbirth. The profiles prompt researchers and clinicians to integrate PFCs as multidimensional rather than isolated problems when investigating and addressing patients’ needs. These new PFC profiles indicate and add value to the need to determine adequate tailored (multidisciplinary) treatment approaches and to include all common PFCs in research and clinical reasoning. Including PFC profiles to clarify etiological and other contributing factors to PFC combinations in pregnancy- and parity-related circumstances may further enhance understanding of their impact on women. This may, ultimately, enhance current preventative and curative treatment approaches to benefit the patients involved.

### The role of menstrual complaints

This study also explored the recording frequencies and associations between PFCs and menstrual complaints to explore the role of hormonal fluctuations during the menstruation cycle on presented PFCs. Menstrual complaints seem predominantly recorded in files from nulliparous patients. While low or absent recordings were expected for pregnant patients, low recordings for parous patients raise questions about the true absence of menstrual complaints. Absent menstruation postpartum is normal for a varying amount of time and during the baby’s breastfeeding period. Alternatively, menstrual complaints may be underreported. PTs might assume absence and exclude menstrual status in intakes with pregnant and newly parous patients. Instead, menstrual complaints may only be spontaneously disclosed when they are the cause of a high level of PFC-related problems. Despite differences in reported menstrual complaints, their regular appearance in PPT patient files supports an association with PFCs, which should be clarified in future research.

### Strengths and limitations

This study’s approach to exploring PFCs in profiles via LCA, rather than identifying isolated primary PFCs or bivariate associations between PFCs, is a strength. It allowed for new, comprehensive insights into co-occurrences between three or more PFCs.

However, this study also has methodological shortcomings. First, the intended stratification and inclusion of patients in PPT practices and the lack of knowledge about the number of pregnant, parous, and nulliparous patients aged 18 to 45 receiving PPT in the Netherlands limits the generalizability of the results. Second, selection bias from PTs self-selecting the included patient files and not inventorying PT specializations affecting the available patients in the PPT practices may have influenced the results. Third, inclusion criteria were relatively stringent. Pregnant patients with children, parous patients with children older than two years, nulliparous patients outside the age range of 18 to 45 years, and postmenopausal patients and patients with somatic and mental comorbidity were all excluded because their characteristics may have distorted the outcomes. Fourth, the retrospective file review design might have limited the accuracy and generalizability of the results. Including explicit input from the selected patients may yield more insight into the accuracy of PT recordings of PFCs and the patients’ self-disclosure level. Given these limitations, prospective research is recommended to address the robustness of the PFC profiles in a more representative and larger group of PTs and PPT patients.

## Conclusion

The identified PFC profiles are a first step toward decreasing the risk of treating PFCs in isolation and further facilitating the inclusion and understanding of etiological factors for combinations of PFCs in scientific research and clinical practice. The new insights may enhance PT’s history-taking, clinical reasoning, and choice of adequate (multidisciplinary) treatment approaches, yielding better treatment outcomes. The PFC profiles may also facilitate patient self-disclosure by understanding that PFCs do not occur in isolation. Discussing PFCs in profiles may reassure patients during psychoeducation, help discuss tailored treatment options, and manage patients’ expectations. Based on the correlations between PFCs and menstrual complaints, hormonal influences on PFCs should not be ignored in research and clinical practice.

## Ethics and consent

The Ethical Review Board of the Open University of the Netherlands approved the study protocol (Date: May 29th, 2019/No. U2019/03973/HVM) in May 2019. All patients provided written informed consent for their data to be included in the online survey conducted by participating PTs.

## Data Availability

OSF: Pelvic Health Problems in Young Adult Women, Doi: https://doi.org/10.17605/OSF.IO/ZG2BN ( https://osf.io/dwny) ^35^ This project contains the following underlying data: Data en Codeboek PPT studie-DEF-DEF.xlsx Function_bootstrap.R Functions_plot.R LCA.R Data are available under the terms of the Creative Commons Zero "No rights reserved" data waiver (CC0 1.0 Public domain dedication) ( http://creativecommons.org/publicdomain/zero/1.0/). OSF: Pelvic Health Problems in Young Adult Women, doi https://doi.org/10.17605/OSF.IO/DWNYH ( https://osf.io/vu2e7) ^36^ Appendix A.pdf Data are available under the terms of the Creative Commons Zero "No rights reserved" data waiver (CC0 1.0 Public domain dedication) ( http://creativecommons.org/publicdomain/zero/1.0/).
