# Supplementary material for: Pelvic floor complaint profiles in young adult women; a file review study in pelvic physical therapy practices in the Netherlands
Source: Open Res Eur. 2026 Jan 21;5:78. Originally published 2025 Mar 24. [Version 2] doi: 10.12688/openreseurope.19785.2 (PMC12757680; doi:10.12688/openreseurope.19785.2)
Supplement: Peer review history

# Reviewer report: Reviewer response for version 2

**DOI:** 10.21956/openreseurope.24699.r68503

**Published:** 2026-02-11

**Authors:** Alime Büyük

**Affiliations:** 1 Akdeniz University, Antalya, Turkey

Thank you so much. Such an amazing work.

If applicable, is the statistical analysis and its interpretation appropriate?

Yes

Is the study design appropriate and is the work technically sound?

Yes

Is the work clearly and accurately presented and does it engage with the current literature?

Yes

Are the conclusions drawn adequately supported by the results?

Yes

Are sufficient details of methods and analysis provided to allow replication by others?

Yes

Are all the source data and materials underlying the results available?

Yes

Reviewer Expertise:

NA

I confirm that I have read this submission and believe that I have an appropriate level of expertise to confirm that it is of an acceptable scientific standard.

# Reviewer report: Reviewer response for version 1

**DOI:** 10.21956/openreseurope.21403.r64596

**Published:** 2026-01-01

**Authors:** Alime Büyük

**Affiliations:** 1 Akdeniz University, Antalya, Turkey

Summary / overall recommendation

 This retrospective file review addresses a clinically important gap: pelvic physical therapy patients frequently present with multiple co-occurring pelvic floor complaints, yet research and (sometimes) documentation can over-focus on a single “main complaint,” which may narrow clinical reasoning and treatment planning. The authors’ use of latent class analysis (LCA) to explore multi-complaint “profiles” is appropriate for hypothesis generation and is potentially useful for intake frameworks and patient education.

 Recommendation: *Minor revisions* (primarily for reporting transparency, interpretability of the LCA outputs, and avoiding etiologic overreach given retrospective documentation bias).

 Minor revisions

1. Correlations section clarity. You describe correlations for dichotomous variables using a specific formula. Name the coefficient explicitly (e.g., phi/tetrachoric rationale) and interpret negative associations cautiously as they may reflect selective documentation.
2. Group definitions and timelines. Eligibility criteria specify parous patients with youngest child ≤2 years and not pregnant again, plus other constraints. Consider adding a short rationale for the 2-year threshold and how “age of youngest child” was used (descriptive only vs analytic).
3. Menstrual complaints in postpartum/parous group. Your Discussion appropriately raises breastfeeding/amenorrhea and underreporting. I’d recommend adding this point earlier (limitations) to avoid readers interpreting low recording as true absence.
4. Results phrasing should align with data type. Replace wording like “complaints were present” with “were recorded,” consistently throughout (fits your retrospective design).

Line-level edits (specific suggestions)

 Abstract (edits to improve precision and reduce over-claim)

 Background

- Change “Based on available evidence, pelvic floor complaint profiles… have not been investigated” → “have not been described using real-world pelvic PT records and multi-complaint clustering methods” (more specific).

Method

- Add: “Complaints were extracted from clinical records and coded as binary (recorded/not recorded).”
- Add: “Model selection used BIC/log-likelihood and expert clinical review.”

Results

- Replace “characterized by” with “showed the highest probabilities for recorded…” (aligns with probabilistic classes and documentation-based data).
- Add numeric class prevalence and the highest item probabilities (once you provide the figure/table).

Conclusion

- Soften causality: “may facilitate the inclusion and consideration of potential contributing factors” rather than “understanding of etiological factors.”

Results section (wording consistency)

- In “Descriptive results,” revise “menstruation is unlikely during pregnancy” sentence to clarify this reflects **record timing artifacts** (pre-pregnancy notes) and treat it as an indicator of documentation limitations.

Pelvic-health clinical interpretation (what this supports now)

 Even with the above limitations, the work supports a practical takeaway: routine pelvic intake should screen across bladder, bowel, POP symptoms, pelvic pain, and sexual pain because co-occurrence is common and may be missed when focusing on a single main complaint.

If applicable, is the statistical analysis and its interpretation appropriate?

Yes

Is the study design appropriate and is the work technically sound?

Yes

Is the work clearly and accurately presented and does it engage with the current literature?

Yes

Are the conclusions drawn adequately supported by the results?

Yes

Are sufficient details of methods and analysis provided to allow replication by others?

Yes

Are all the source data and materials underlying the results available?

Yes

Reviewer Expertise:

Pelvic floor physiotherapy, chronic pelvic pain, pelvic floor dysfunctions, myofascial trigger point release

I confirm that I have read this submission and believe that I have an appropriate level of expertise to confirm that it is of an acceptable scientific standard, however I have significant reservations, as outlined above.

## References

[1] : Effects of positive sex education and pelvic floor strengthening program on sexual and pelvic health among youngest old: a randomized controlled trial. *BMC Geriatrics* .2025;25(1) : 10.1186/s12877-025-06152-9 10.1186/s12877-025-06152-9 40611002 PMC12224779

# Response

**Published:** 2026-01-14

**Authors:** Alma Brand

**Affiliations:** Psychology, Open Universiteit, Heerlen, Limburg, The Netherlands

Thank you for your compliments, suggestions, and recognition of our work. We have addressed your feedback as follows:

1. Correlation section: The φ-coefficient is now named explicitly, and we have phrased the interpretation of the negative correlation more carefully. Thank you for pointing this out.
2. Group definitions and timelines: A short rationale was added to explain the inclusion criteria. The descriptive character of “age younger than two” has been clarified.
3. Menstrual complaints in the postpartum/parous group: We added a remark on this topic in the results section, anticipating discussion of this finding in the discussion.
4. Thank you for this observation. The wording has been consistently aligned with the data type.

Edits to abstract, result section: The suggested changes to the abstract have been made. Thank you for pointing these out. The class prevalence and highest item probabilities have been added to the results in the section after Figure 1. The wording in the descriptive results has been consistently revised concerning the record-timing artefacts. We hope to have addressed your feedback and suggestions to your satisfaction.

# Reviewer report: Reviewer response for version 1

**DOI:** 10.21956/openreseurope.21403.r64600

**Published:** 2025-12-30

**Authors:** Pauliina Aukee

**Affiliations:** 1 Wellbeing Services County of Central Finland, Jyväskylä, Finland

The idea of the article is unique and should that's why to be published. However clinical practices may differ in other countries. It's unclear to a reader, do patient's go straight to visit ft or after doctor's recomendation? Why any qualified questionairs were not used at the beginning of ft-visit. 

 Did the patients report present, past or continuous symptoms? 

 There might be a selection bias since ft:s selected the cases. Did they have a rule to select in certain order or during certain time period.

If applicable, is the statistical analysis and its interpretation appropriate?

Yes

Is the study design appropriate and is the work technically sound?

Yes

Is the work clearly and accurately presented and does it engage with the current literature?

Yes

Are the conclusions drawn adequately supported by the results?

Yes

Are sufficient details of methods and analysis provided to allow replication by others?

Yes

Are all the source data and materials underlying the results available?

Partly

Reviewer Expertise:

Urogynecology

I confirm that I have read this submission and believe that I have an appropriate level of expertise to confirm that it is of an acceptable scientific standard.

# Response

**Published:** 2026-01-14

**Authors:** Alma Brand

**Affiliations:** Psychology, Open Universiteit, Heerlen, Limburg, The Netherlands

Thank you for your compliments. In the Netherlands, access to (pelvic) physical therapy is provided upon a doctor’s referral, and direct access is possible. When directly accessing a PT, a screening is conducted to determine whether the complaints align with the typical patterns treated. If so, treatment is started, and the patient’s GP is informed. If this is not the case, the patient is advised to consult their GP to determine if treatment can be started. The GP may also decide to refer the patient to other specialists for further examination. In our limitations section, we have addressed the characteristics of the Dutch PPT system compared to those of other countries. PTs have access to valid and reliable questionnaires and may choose to use them. They are trained to always take an extensive biopsychosocial history at the start of each treatment period and document it in their patient files. In our survey, we asked PTs to record all PFCs documented in their patient files to identify combinations of PFCs. During treatment, PFCs may be resolved in stages, so the documented PFCs were past, present, or ongoing. We are aware of the potential selection bias. PTs were instructed to select patients who met the inclusion criteria, were currently being treated, or had been under treatment in the year before participation.

# Reviewer report: Reviewer response for version 1

**DOI:** 10.21956/openreseurope.21403.r56177

**Published:** 2025-08-13

**Authors:** Jie Hao

**Affiliations:** 1 Southeast Colorado Hospital, Springfield, Colorado, USA

The manuscript entitled "Pelvic floor complaint profiles in young adult women: a file review study in pelvic physical therapy practices in the Netherlands" has been reviewed. This manuscript presents a valuable exploratory investigation into pelvic floor complaint profiles among young adult women. The application of latent class analysis to define pelvic floor complaint profiles is novel and appropriate. Several issues need to be addressed to ensure scientific rigor and the validity of the findings.

 The use of retrospective documentation from PTs introduces variability in completeness and accuracy. The absence of direct patient-reported data could result in underreporting of sensitive symptoms like sexual dysfunction or incontinence. The authors should consider acknowledging this limitation more clearly and suggest prospective studies using standardized patient questionnaires.

 While presence/absence of symptoms is recorded, the lack of data on severity, duration, or functional impact of each PFC limits the clinical utility of the identified profiles.

 The findings may be highly specific to the Dutch PPT context. The authors should discuss how differences in training, practice scope, or healthcare systems in other countries might limit generalizability.

If applicable, is the statistical analysis and its interpretation appropriate?

Partly

Is the study design appropriate and is the work technically sound?

Partly

Is the work clearly and accurately presented and does it engage with the current literature?

Yes

Are the conclusions drawn adequately supported by the results?

Yes

Are sufficient details of methods and analysis provided to allow replication by others?

Partly

Are all the source data and materials underlying the results available?

Yes

Reviewer Expertise:

Physical therapy, rehabilitation

I confirm that I have read this submission and believe that I have an appropriate level of expertise to confirm that it is of an acceptable scientific standard, however I have significant reservations, as outlined above.

# Response

**Published:** 2026-01-14

**Authors:** Alma Brand

**Affiliations:** Psychology, Open Universiteit, Heerlen, Limburg, The Netherlands

Thank you for your compliments and constructive feedback. We have addressed your comments as follows:

**Comment 1 (Retrospective data)**: We have acknowledged the limitation you highlighted more clearly and included your suggestion about prospective studies.

**Comment 2 (Lack of data on severity, duration, functional impact):** Thank you for pointing this out. This point has been added as a fifth limitation. Our priority was to demonstrate that PFC profiles exist and are clinically relevant. Now that we have shown this, the next step would be to include severity, duration, and functional impact in the analyses to assess the clinical utility of the profiles further.

**Comment 3 (Dutch context):** Thank you for pointing this out. This point has been added as a sixth limitation. You are correct in pointing out that the standard of PPT care in the Netherlands is high. Higher than in many other countries, and therefore, the findings may not be generalizable to other countries. I would like to explore this to determine if the profiles are universal. We hope we have addressed your concerns satisfactorily.
